# Ethnicity matching and outcomes after kidney transplantation in the United Kingdom

**DOI:** 10.1371/journal.pone.0195038

**Published:** 2018-04-13

**Authors:** Bhavini Pisavadia, Adam Arshad, Imogen Chappelow, Peter Nightingale, Benjamin Anderson, Jay Nath, Adnan Sharif

**Affiliations:** 1 Department of Nephrology and Transplantation, Queen Elizabeth Hospital, Edgbaston, Birmingham, United Kingdom; 2 University of Birmingham, Birmingham, United Kingdom; 3 Department of Medical Statistics, Queen Elizabeth Hospital, Edgbaston, Birmingham, United Kingdom; 4 School of Immunology and Immunotherapy, University of Birmingham, Birmingham, United Kingdom; Medizinische Universitat Graz, AUSTRIA

## Abstract

**Background:**

Kidneys from non-white donors have inferior outcomes, but it is unclear if ethnicity matching between donors and recipients achieves better post kidney transplant outcomes.

**Methods:**

We undertook a retrospective, population cohort study utilising UK Transplant Registry data. The cohort comprised adult, kidney-alone, transplant recipients receiving their first kidney transplant between 2003–2015, with data censored at 1^st^ October 2016. We included 27,970 recipients stratified into white (n = 23,215), black (n = 1,679) and south Asian (n = 3,076) ethnicity, with median post-transplant follow-up of 1,676 days (IQR 716–2,869 days). Unadjusted and adjusted Cox regression survival analyses were performed to investigate ethnicity effect on risk for graft loss and mortality.

**Results:**

In unadjusted analyses, matched ethnicity between donors-recipients resulted in better outcomes for delayed graft function, one-year creatinine, graft and patient survival but these differed by ethnicity matches. Compared to white-to-white transplants, risk for death-censored graft loss was higher in black-to-black and similar among Asian-to-Asian transplants, but mortality risk was lower for both black-to-black and Asian-to-Asian transplants. In Cox regression models, compared to white donors, we observed higher risk for graft loss with both south Asian (HR 1.38, 95%CI 1.12–1.70, p = 0.003) and black (HR 1.66, 95%CI 1.30–2.11, p<0.001) donated kidneys independent of recipient ethnicity. We observed no mortality difference with south Asian donated kidneys but increased mortality with black donated kidneys (HR 1.68, 95%CI 1.21–2.35, p = 0.002). Matching ethnicities made no significant difference in any Cox regression model. Similar results were observed after stratifying our analysis by living and deceased-donor kidney transplantation.

**Conclusions:**

Our data confirm inferior outcomes associated with non-white kidney donors for kidney transplant recipients of any ethnicity in a risk-adjusted model for the United Kingdom population. However, contrary to non-renal transplant literature, we did not identify any survival benefits associated with donor-recipient ethnicity matching.

## Introduction

Minority ethnics in the United Kingdom comprise a third of patients on the national deceased-donor kidney waiting list and have longer waits for an adult kidney transplant; median wait of 1070 days (south Asians) and 1134 days (Blacks) compared to 882 days (Whites) [1]. Biological differences between ethnic groups, such as disparate frequencies of different blood groups and particular combinations of HLA alleles, contributes to these prolonged waiting time as minority ethnic recipients are waiting to receive kidney allografts from a predominantly white deceased donor organ pool. While this delay can be overcome with a suitable live kidney donor, this may not be available for all kidney transplant candidates.

Biological differences between ethnic groups partly influences prolonged waiting times but it is unclear if they can also influence kidney transplant outcomes. In non-renal solid organ transplantation, published studies suggest transplanting recipients with ethnically matched donors provides the best outcomes after heart [2], lung [3] and liver [4] transplantation. Data from the kidney transplantation literature is limited but Locke and colleagues recently published data suggesting kidneys donated after cardiac (but not brain) death from Black donors were associated with the best patient and graft survival for Black recipients [5]. This contrasts with evidence from registry data that kidneys donated by minority ethnics (especially Black individuals) are associated with poorer graft survival for any kidney transplant recipient [6–8]. Brown and colleagues have also reported their single-center experience of outcomes in black recipients receiving black (n = 35) versus white (n = 150) deceased-donor kidneys and found similar patient survival but increased risk for graft loss with black-to-black deceased donor kidney transplantation [9].

Current data is conflicting but also irrelevant to the United Kingdom cohort of patients. Firstly, minority ethnic demographics differ between the United Kingdom and the United States. Secondly, Black individuals in the United Kingdom do not share the same genetic risk variants for kidney disease (e.g. APOL1) as African Americans, which could explain some of the adverse outcomes observed with Black donors in the United States [10]. Therefore, the aim of this population-cohort study was to test the hypothesis that ethnicity matching achieves better post kidney transplant outcomes in a contemporary cohort of patients in the United Kingdom.

## Subjects and methods

### Study population

The UK Transplant Registry (UKTR) is held by National Health Service Blood and Transplant (NHSBT), and all 23 UK adult kidney transplant centers provide mandatory data to this registry. We retrospectively analyzed data for all adult (aged 18 and over) kidney-alone allograft recipients who received their first transplant between April 1st 2003 and March 31st 2015 (data censored at 1^st^ April 2017). Repeat transplants for the same patient were excluded from analysis. Our study population initially included 29,142 recipients who underwent kidney transplantation within those dates. As our analysis was focused on the three main ethnicity groups, we excluded 1,172 cases due to ethnicity being documented as other or unknown for either donor or recipient. This left a study cohort of 27,970 where donor or recipient ethnicity was white, black or south Asian (also termed Indo-Asian). Missing data for donor and recipient ethnicity constituted 0.1% and 0.5% respectively. Data analysis indicated this was not significantly different from the characteristics of donors and recipients with ethnicity data and was thus assumed to be missing at random.

### Study variables

Variables were obtained from the UK Transplant Registry and the majority were inputted directly for analysis. Ethnicity was defined by specialist nurses for organ donation and transplant coordinators for donors and recipients respectively. We stratified cause of end-stage kidney disease into; diabetes, hypertension, glomerulonephritis, polycystic kidney disease and other. HLA mismatch was categorized into four levels of matching, as utilized by NHS Blood and Transplant for allocation purposes; level 1 (HLA mismatch 0), level 2 (HLA mismatch 0 DR and 0/1 B), level 3 (HLA mismatch 0 DR and 2B, or 1 DR and 0/1 B), and level 4 (1 DR and 2B, or 2 DR). We classified sensitization as any recipient with a calculated reaction frequency (CRF) greater than 0%.

### Outcome measures

The primary outcome measure was death-censored graft survival after kidney transplantation, with patient survival our secondary outcome. In addition, we analyzed variables related to graft function including delayed graft function (defined as need for dialysis within first week after kidney transplantation) and one-year creatinine. We also undertook sensitivity analyses to stratify results based upon donor versus recipient ethnicity, focusing on our primary and secondary outcomes measures.

### Statistical analysis

Initially a range of variables were compared across ethnic groups. Continuous and ordinal variables were analyzed using Mann-Whitney, with Fisher’s exact test used for dichotomous variables, or Kruskal-Wallis tests with more than two groups (with Shaffer correction). Univariable survival analysis was then performed, using Cox regression models, with Kaplan-Meier curves to visualize the results.

We developed Cox proportional hazards models for estimation of kidney allograft loss or death using indicator variables for donor and recipient ethnicity, with proportionality assumption checked for each variable and the whole model (using scaled Schoenfeld Residuals). Complete case only analysis was undertaken for multiple regression, with data censored for event occurrence or study end date (1^st^ April 2017). The variables included in the models were based on clinical judgment and adjusted for donor, recipient and graft characteristics that are known to be predictors of long-term outcomes including; donor age, donor smoker, donor sex, donor ethnicity, recipient age, recipient sex, recipient ethnicity, recipient diabetes, waiting time, cold ischemic time, sensitization (calculated reaction frequency), HLA mismatch, delayed graft function, rejection, type of donor and ethnicity matching between donors/recipients. Before we included these variables, we undertook exploratory data analysis to identify interactions between model covariates to improve the model fit. All analyses were performed using IBM SPSS 22 (IBM Corp. Armonk, NY), with p<0.05 deemed to be indicative of statistical significant throughout. All p values are uncorrected in the context of subgroup analyses.

### Approvals

NHS Blood and Transplant holds the database for transplantation in the UK but they had no role in the study design, data analysis, data interpretation, or writing of this report. The corresponding author had full access to all data. This project was registered as an audit with University Hospitals Birmingham NHS Trust (audit identifier; CARMS-12578).

## Results

### Demographics

A total of 27,970 kidney transplant recipients were available for analysis after exclusions. Baseline variables of recipients stratified by ethnicity are highlighted in Table 1. Stratified into our three main ethnicity groups, we had 23,215, 1,679 and 3,076 kidney transplant recipients of white, black and south Asian ethnicity respectively. Median follow up time from kidney transplantation was 1,676 days (IQR 716 to 2,869 days).

**Table 1:** Demographic characteristics of kidney transplant recipients.

Variable		White recipient	Black recipient	South Asian recipient	P value
Number (%)		23,215 (83.0%)	1,679 (6.0%)	3,076 (11.0%)	-
***RECIPIENT-RELATED VARIABLES***					
Age (yrs, mean±SD)		48.1±13.9	47.0±12.2	47.5±13.4	<0.001
Male gender		61.6%	60.7%	61.8%	0.733
Cause of end-stage kidney failure	Diabetes	9.3%	14.6%	25.4%	<0.001
	GN^1^	33.3%	26.3%	32.9%	
	Hypertension	6.1%	28.7%	10.6%	
	PKD	19.9%	9.7%	18.0%	
	Other	31.5%	20.8%	25.4%	
HLA mismatch	Level 1	14.7%	6.1%	6.4%	<0.001
	Level 2	26.6%	28.3%	29.9%	
	Level 3	43.6%	52.7%	49.1%	
	Level 4	15.1%	12.9%	14.6%	
Blood group	O	44.0%	50.6%	32.0%	<0.001
	A	43.1%	27.2%	25.6%	
	B	9.2%	17.9%	32.4%	
	AB	3.7%	4.2%	10.0%	
CMV serostatus +		43.7%	75.6%	78.6%	<0.001
Sensitized (CRF >0%)		33.9%	31.6%	29.3%	<0.001
Body mass index (mean±SD)		26.3±4.7	26.5±5.0	25.6±4.5	<0.001
***DONOR-RELATED VARIABLES***					
Blood group	O	51.6%	56.8%	41.2%	<0.001
	A	39.3%	26.3%	26.8%	
	B	6.8%	14.2%	25.3%	
	AB	2.2%	2.8%	6.7%	
Male gender		50.4%	53.2%	52.1%	0.016
Ethnicity	White	97.8%	71.9%	72.7%	<0.001
	Black	0.6%	22.2%	1.9%	
	South Asian	1.0%	3.3%	23.1%	
Age (years, mean±SD)		48.6±14.4	46.5±15.4	47.2±15.1	<0.001
Donor diabetes		5.9%	6.2%	6.6%	0.380
Donor hypertension		25.3%	27.0%	27.0%	0.120
Donor smoking exposure		44.9%	44.3%	46.1%	0.445
Body mass index (mean±SD)		26.5±4.8	26.5±4.9	26.3±4.5	0.056
CMV serostatus +		47.3%	57.7%	58.4%	<0.001
Donor type	Live	37.5%	25.7%	25.1%	<0.001
	DBD^2^	42.1%	51.5%	51.2%	
	DCD^3^	20.4%	22.7%	23.7%	
***TRANSPLANT-RELATED VARIABLES***					
Antibody incompatible	ABO	1.8%	0.1%	0.2%	<0.001
	HLA	2.0%	0.2%	0.3%	
Waitlist time (days, median [IQR])		588 [221–1206]	1015 [504–1578]	861 [367–1572]	<0.001
Cold ischemic time (mins, median [IQR])		733 [217–1022]	820 [454–1071]	810 [431–1064	<0.001

^1^Glomerulonephritis

^2^Donation after brain death

^3^Donation after cardiac death

Deceased-donor kidney transplants comprised 64.5% of the cohort. Black and south Asian donors comprised bigger proportions as live-donors (3.8% versus 6.9% respectively) compared to deceased-donors (1.1% and 1.7% respectively). Overall, total kidney donors of black ethnicity and south Asian ethnicity were more likely to have diabetes than white donors (12.1% versus 17.4% versus 5.7% respectively, p<0.001), more likely to have hypertension (29.1% versus 34.2% versus 25.6% respectively, p = 0.001), more likely to be younger in mean years (40.3±13.1 versus 43.9±13.4 versus 48.7±14.6 respectively, p<0.001) but less likely to have smoking history (39.6% versus 24.4% versus 45.6% respectively, p<0.001). However, as shown in Table 1, there was no significant difference in the distribution of these donors stratified by recipient ethnicity. The characteristics of donors stratified by ethnicity is summarized in Table 2.

**Table 2:** Donor characteristics stratified by ethnicity.

Variable		White donor	Black donor	South Asian donor	P value
Number (%)		26403 (94.4%)	575 (2.1%)	992 (3.5%)	-
Donor	Live	8867 (89.3% of all live donors)	377 (3.8% of all live donors)	685 (6.9% of all live donors)	<0.001
	Deceased	17536 (97.2% of all deceased donors)	198 (1.1% of all deceased donors)	307 (1.7% of all deceased donors)	
Proportion of live donors (%)		8867 (89.3%)	377 (3.8%)	685 (6.9%)	<0.001
Proportion of deceased donors (%)		17539 (97.2%)	198 (1.1%)	307 (1.7%)	<0.001
Age (mean±SD)		48.7±14.6	40.3±13.1	43.9±14.6	<0.001
Diabetes (%)		1505 (5.7%)	70 (12.1%)	173 (17.4%)	<0.001
Hypertension (%)		6760 (25.6%)	167 (29.1%)	339 (34.2%)	0.001
Smoking history (%)		12041 (45.6%)	228 (39.6%)	242 (24.4%)	<0.001

### Graft survival

**Fig 1:**
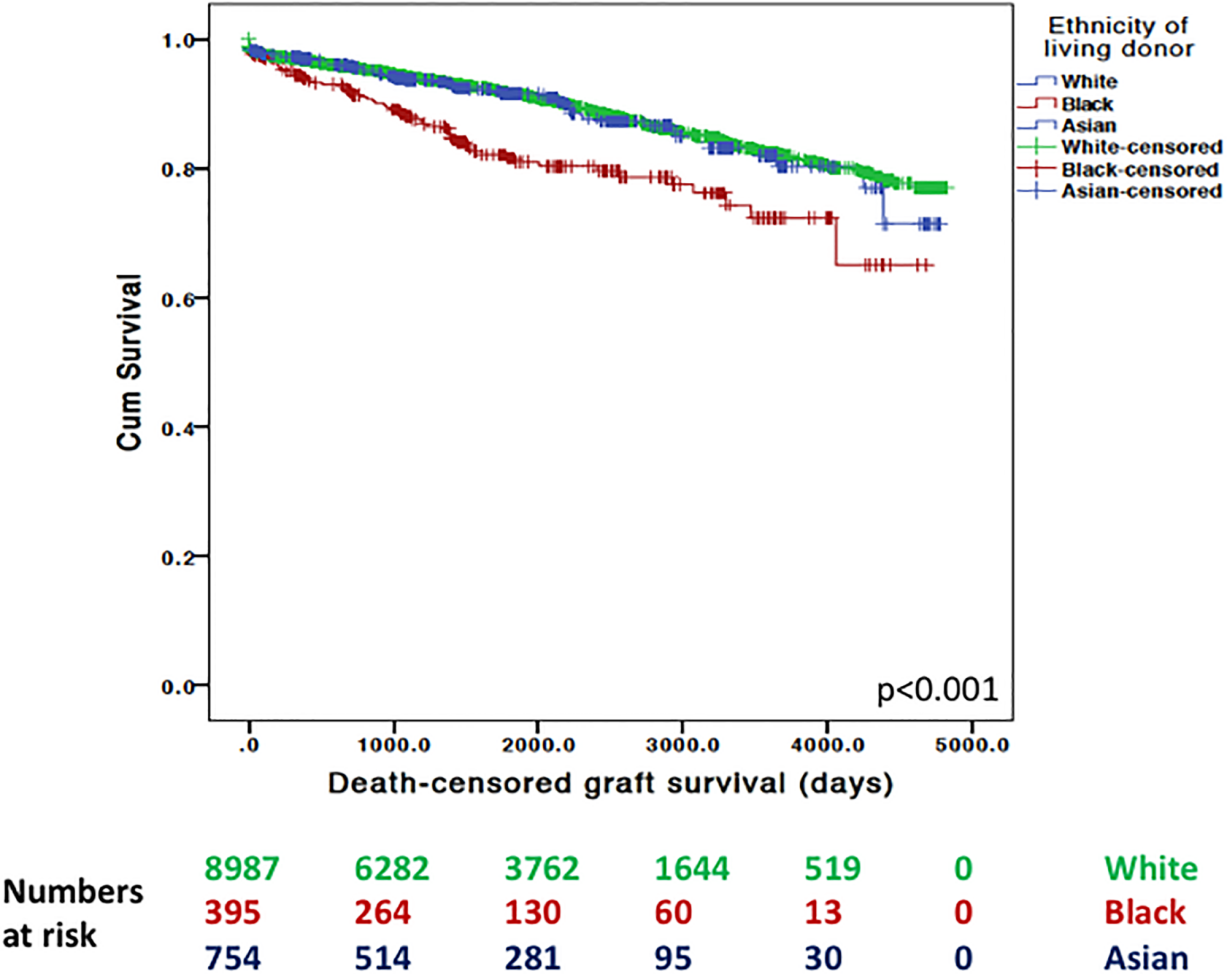
Unadjusted Kaplan-Meier survival plots of death-censored graft survival after living kidney donation based upon donor ethnicity.

**Fig 2:**
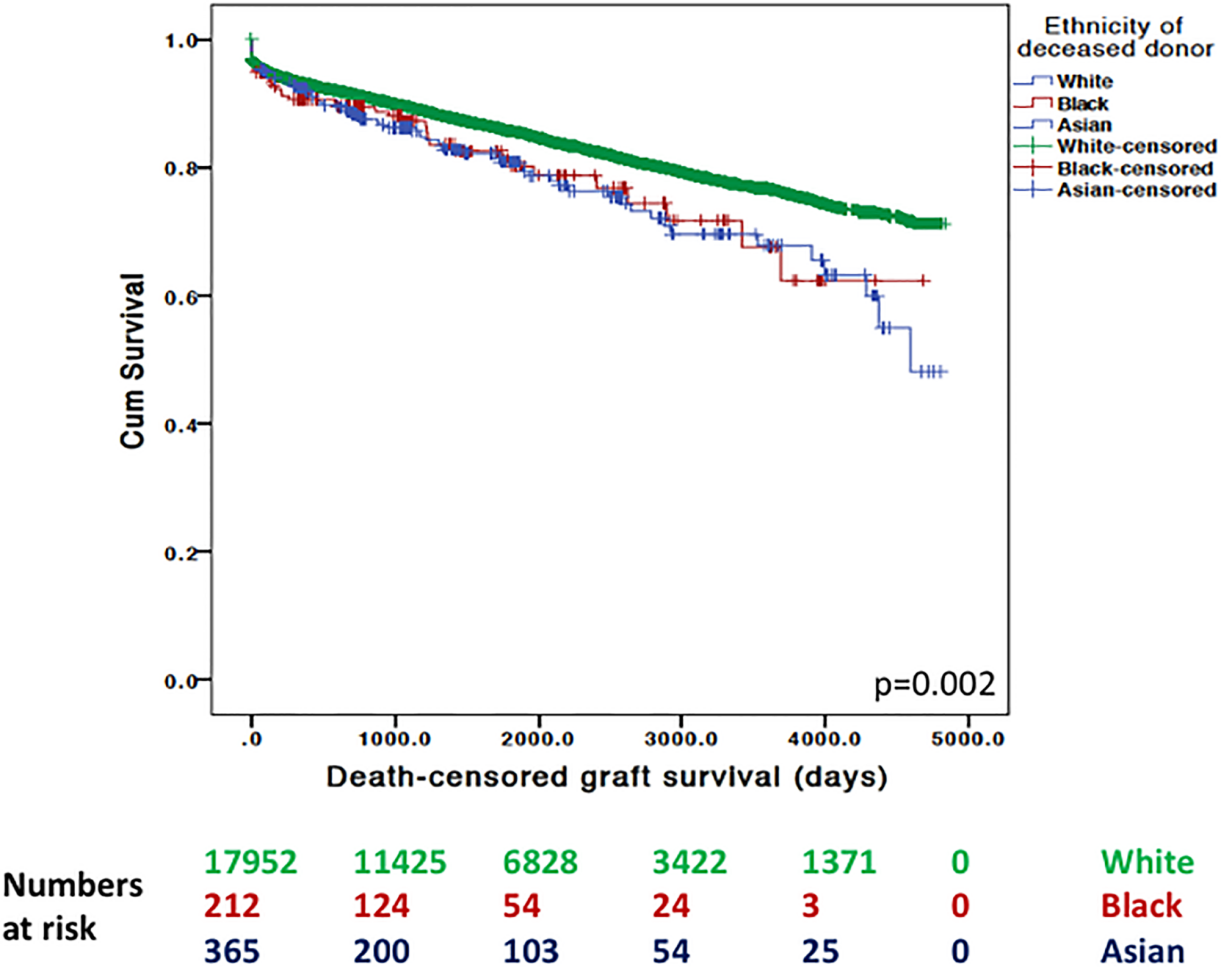
Unadjusted Kaplan-Meier survival plots of death-censored graft survival after deceased kidney donation based upon donor ethnicity.

Percentage of graft survival is given until the event had occurred or the study ended. In our unadjusted analyses, death-censored graft survival was different between recipient ethnic groups (white = 87.0%, black = 82.8%, south Asian = 86.7%, p<0.001). In addition, we identified a significant difference in death-censored graft survival for recipients receiving kidneys stratified by donor ethnic groups (white = 86.8%, black = 82.8%, south Asian = 86.9%, p<0.001). This remained the case whether donors were after living (see Fig 1) or deceased (see Fig 2) kidney donation.

Looking at causes of graft loss, we observed significant differences in cause of death-censored graft loss stratified by recipient ethnicity (p = 0.016). Any type of rejection as the cause for graft loss among white recipients occurred in 37.0% of case, followed by vascular events (12.3%), recurrent disease (6.0%) or other (44.2%). For black recipients, any type of rejection was the underlying etiology in 31.8% of cases followed by vascular events (10.5%), recurrent disease (4.7%) or other (52.4%). In south Asian recipients, any type of rejection was the cause of graft loss in 35.7% in cases followed by vascular events (15.0%), recurrent disease (4.1%) and other (46.6%).

We did not identify any significant differences in cause of graft loss among any kidney transplant patients’ dependent upon donor ethnicity.

### Patient survival

Percentage of patient survival is given until the event had occurred or the study ended. In our unadjusted analysis, patient survival was not significantly different between recipient ethnic groups (white = 88.6%, black = 91.9%, south Asian = 89.9%, p = 0.140) regardless of donor ethnicity. However, we did identify a significant difference in patient survival for recipients of any ethnicity receiving kidneys stratified by donor ethnic groups (white = 88.7%, black = 91.7%, south Asian = 93.2%, p = 0.005).

Looking at causes of death, there was significant differences in etiology among recipient ethnicity groups (p = 0.002). Among white transplant recipients, cases of death were classified as; cardiovascular events (20.3%), cerebrovascular events (3.5%), infection (28.1%), cancer (22.5%) and other (25.6%). For black transplant recipients, cases of death were classified as; cardiovascular events (23.3%), cerebrovascular events (3.5%), infection (23.3%), cancer (17.4%) and other (32.6%). Finally, among south Asian transplant recipients, cases of death were classified as; cardiovascular events (19.9%), cerebrovascular events (2.6%), infection (43.9%%), cancer (12.2%) and other (21.4%).

We did not identify any significant differences in cause of death among any kidney transplant patients’ dependent upon donor ethnicity.

### Functional graft outcomes

We observed higher rates of delayed graft function among black (31.6%) and south Asian recipients (23.2%) compared to white recipients (19.1%), regardless of donor ethnicity (p<0.001). In contrast, we observed lower rates of delayed graft function in any recipient receiving kidneys from black (14.5%) or south Asian (12.8%) donors compared to white donors (20.7%) regardless of recipient ethnicity (p<0.001).

After stratification into donor type, we observed delayed graft function rates after live kidney donation of 5.5%, 9.0% and 5.6% for recipients receiving kidneys from white, black and south Asian donors respectively (p = 0.011). With donation after brain death, we observed no difference in delayed graft function rates of 22.8%, 19.0% and 21.9% for recipients receiving kidneys from white, black and south Asian donors respectively (p = 0.814). Similarly, we observed no difference in delayed graft function rates of 39.8%, 46.3% and 43.0% for recipients receiving kidneys donated after cardiac death from white, black and south Asian donors respectively (p = 0.652). However, numbers for the latter were very small to be statistically meaningful.

Repeating this analysis for recipient ethnicity, we observed delayed graft function after live kidney donation of 5.4%, 8.3% and 6.3% for white, black and south Asian recipients respectively regardless of donor ethnicity (p = 0.043). With donation after brain death, we observed significant difference in delayed graft function rates of 22.0%, 33.8% and 22.0% for white, black and south Asian recipients respectively (p<0.001). Similarly, we observed significant difference in delayed graft function rates of 38.2%, 53.2% and 43.5% for kidneys donated after cardiac death for white, black and south Asian recipients respectively (p<0.001).

Median [+ interquartile range] one-year creatinine among surviving kidneys was significantly different between recipients receiving kidneys from white (127 [104–158] mmol/l), black (127 [103–161] mmol/l) and south Asian (120 [97-147mmol/l) donors (p<0.001). Median [+ interquartile range] one-year creatinine among recipients with surviving kidneys was higher among black recipients (142 [115–180] mmol/l) versus white (127 [105–158] mmol/l) or south Asian recipients (116 [94–145] mmol/l) regardless of donor ethnicity (p<0.001).

### Sensitivity analyses stratified by ethnicity

Firstly, we stratified all kidney transplants into ethnically matched combinations. Ethnically matched kidney transplantation occurred in the vast majority of live donor transplantation, accounting for 85.0% (black-to-black), 92.0% (south Asian-to-south Asian) and 92.0% (white-to-white) of total numbers of live donor kidney transplantation for that particular ethnicity group. However, in the context of deceased donor kidney transplantation, ethnically matched kidney transplantation dropped significantly for black-to-black (32.2%) and south Asian-to-south Asian (36.4%) compared to white-to-white (81.2%) from total number of deceased donor kidney transplantation for that particular ethnicity group.

In Table 3, we display the unadjusted hazard ratios for risk of graft loss among ethnically matched kidney transplants. Compared the reference (white-to-white), our analyses demonstrated significant heterogeneity with regards to outcomes associated with ethnically matched kidneys. While the risk for graft loss was general higher for all ethnicity combination kidney transplants compared to white-to-white (exception being south Asian-to-south Asian), we observed a wide array of results for mortality. In these unadjusted analyses, ethnically matched transplants between minority ethnics appeared to have reduced risk for mortality compared to the reference white-to-white group, while some combinations between minority ethnic groups resulted in increased risk for mortality (e.g. black-to-south Asian) but numbers were small for many of these combinations which may skew the results.

**Table 3:** Unadjusted analyses of risk for graft loss and mortality stratified by donor-to-recipient ethnicity matching.

Donor to Recipient Ethnicity	n	Graft loss			Mortality		
		HR (95% CI)	Events	p value	HR (95% CI)	Events	p value
Asian to Asian	734	0.95 (0.76–1.18)	83	0.62	0.65 (0.48–0.87)	44	0.004
Asian to Black	50	1.86 (1.03–3.36)	11	0.040	0.43 (0.11–1.73)	2	0.24
Asian to White	196	1.42 (1.00–2.03)	31	0.051	0.88 (0.53–1.46)	15	0.61
Black to Asian	62	1.46 (0.76–2.81)	9	0.26	2.04 (1.02–4.09)	8	0.044
Black to Black	387	1.48 (1.16–1.89)	66	0.002	0.67 (0.44–1.01)	23	0.057
Black to White	132	1.33 (0.86–2.06)	20	0.21	1.08 (0.61–1.90)	12	0.79
White to Asian	2280	1.26 (1.12–1.41)	316	<0.001	1.22 (1.07–1.40)	229	0.004
White to Black	1242	1.56 (1.36–1.79)	210	<0.001	0.97 (0.79–1.18)	99	0.75
White to White	22887	1.00	2961	-	1.00	2203	-

### Multivariate regression models

To assess the independent effect of donor versus recipient ethnicity on survival outcomes after kidney transplantation, we undertook Cox proportional hazards models after adjustment for donor, recipient and graft characteristics. The results of the Cox regression models looking at the impact of ethnicity for risk of graft loss and death are shown in Tables 4 and 5 respectively stratified into deceased and living donors. The data shows kidneys from any black donors, whether living or deceased, was associated with an increased risk for graft loss or death for their recipients, while kidneys from deceased-donor south Asians had an increased risk for graft loss for any recipient. Importantly, and contrary to our unadjusted analyses, ethnically matched kidneys were no longer significant in our regression model.

**Table 4:** Multivariate regression model for the risk of graft loss.

*DECEASED KIDNEY DONORS*				
**Parameter**		**Risk for graft loss (HR)**	**95% CI**	**P value**
Donor ethnicity	White	1.00 (reference)	-	-
	Black	1.40	0.99–1.97	0.055
	South Asian	1.57	1.21–2.03	<0.001
Recipient ethnicity	White	1.00 (reference)	-	-
	Black	1.34	1.04–1.74	0.025
	South Asian	1.09	0.86–1.39	0.468
Matched ethnicity		1.06	0.84–1.33	0.648
*LIVING KIDNEY DONORS*				
**Parameter**		**Risk for graft loss (HR)**	**95% CI**	**P value**
Donor ethnicity	White	1.00 (reference)	-	-
	Black	1.86	1.00–3.45	0.050
	South Asian	0.87	0.45–1.69	0.689
Recipient ethnicity	White	1.00 (reference)	-	-
	Black	1.29	0.69–2.39	0.429
	South Asian	1.48	0.78–2.82	0.228
Matched ethnicity		1.07	0.65–1.78	0.789

Variables in model; donor ethnicity, recipient ethnicity, matched ethnicity, recipient age, recipient diabetic (yes/no), HLA mismatch (1, 2, 3, 4), sensitized (CRF>0%), donor sex, donor age, donor smoking status, time on waiting list, cold ischaemic time.

**Table 5:** Multivariate regression model for the risk of death.

*DECEASED KIDNEY DONORS*				
**Parameter**		**Risk for mortality (HR)**	**95% CI**	**P value**
Donor ethnicity	White	1.00 (reference)	-	-
	Black	1.59	1.04–2.43	0.032
	South Asian	0.73	0.47–1.14	0.161
Recipient ethnicity	White	1.00 (reference)	-	-
	Black	1.07	0.73–1.55	0.730
	South Asian	1.16	0.81–1.66	0.409
Matched ethnicity		1.21	0.85–1.70	0.288
*LIVING KIDNEY DONORS*				
**Parameter**		**Risk for mortality (HR)**	**95% CI**	**P value**
Donor ethnicity	White	1.00 (reference)	-	-
	Black	5.13	1.66–15.82	0.004
	South Asian	0.83	0.26–2.61	0.749
Recipient ethnicity	White	1.00 (reference)	-	-
	Black	0.30	0.09–0.98	0.045
	South Asian	1.46	0.48–4.47	0.506
Matched ethnicity		1.41	0.50–3.96	0.520

Variables in model; donor ethnicity, recipient ethnicity, matched ethnicity, recipient age, recipient diabetic (yes/no), HLA mismatch (1, 2, 3, 4), sensitized (CRF>0%), donor sex, donor age, donor smoking status, time on waiting list, cold ischaemic time.

## Discussion

This analysis is the first contemporary report of post kidney transplant outcomes based upon ethnicity matching after kidney transplantation. In our population-cohort analysis of 27,970 patients, we observed disparate outcomes for graft loss and mortality dependent upon donor versus recipient ethnicity. In our adjusted Cox regression models, we found kidneys from black donors were associated with an increased risk for graft loss and death for their recipients, while kidneys from south Asians had an increased risk for graft loss for any recipient only. However, contrary to our unadjusted analyses, we did not identify any difference in risk for graft loss or death in adjusted Cox regression models after ethnicity matched living or deceased-donor kidney transplantation.

Donor-recipient ethnicity matching and post-transplant outcomes has been reported in a few settings after non-renal solid organ transplantation, with outcomes deemed to be better if donor and recipient ethnicities are matched. Kanter and colleagues, in a single-center analysis of 169 consecutive pediatric heart transplant patients, observed inferior graft survival for black versus white recipients and found donor-recipient ethnicity mismatch was predictive of poorer graft survival (5-year graft survival 72.3% versus 48.9% for matched versus mismatched respectively, p = 0.0032) [2]. In a multivariable Cox proportional hazards regression model, donor-recipient ethnicity mismatch was a strong predictor for graft failure (Hazard Ratio 2.137, 95% CI 1.054–4.335) [2]. However, HLA matching could be the confounder in this analysis as black recipients in this series had a higher incidence of a positive retrospective crossmatch compared with white recipients (43% versus 29% respectively, p = 0.053). In the setting of lung transplantation, Allen and colleagues analyzed 11,323 primary lung transplant recipients between 1997 and 2007 from the UNOS data set and found ethnicity matching was associated with reduced risk for mortality [3]. In a risk-adjusted model, cumulative mortality risk was reduced with donor-recipient ethnicity matching (Hazard Ratio 0.88; 95% CI 0.80–0.96, p = 0.006) [3]. Finally, Pang and colleagues identified hepatitis C positive (but not negative) black recipients who underwent liver transplantation had improved graft survival if their donors were black [4].

In the setting of kidney transplantation, Locke and colleagues analyzed a more recent cohort of 25,251 Black transplant recipients between 1993 and 2006 and observed DCD kidneys from black donors facilitated the best graft survival for black recipients in a multivariate regression model (HR 0.43, 95% CI 0.22–0.82, p<0001) [5]. However, in all other scenarios the risk for graft loss was greater and the risk for mortality was greatest for black patients receiving black extended criteria donor kidneys [5]. While this may simply represent statistical aberrations, although additional sensitivity analyses supported their original conclusion, there are plausible explanations for disparate physiological response to brain versus cardiac death that may be modified by ethnicity [11–12]. While our data failed to show any interaction between ethnicity and type of deceased donor (data not shown), our small numbers will likely be susceptible to a type 2 statistical error.

Our data contrasts with non-renal solid organ transplant literature regarding outcomes associated with ethnically matched transplants and the explanation likely relates to our findings, consistent with existing literature, of inferior outcomes associated with non-white donated kidneys. This effect was clearly demonstrated in our analysis but has been shown in other population cohort analyses. For example, Chakkera and colleagues analyzed 79,361 patients from the United States Renal Data System (USRDS) between 1991–2000 and found African-American donors (compared to White) resulted in an increased risk for allograft failure in un-adjusted analyses (Relative Risk 1.28, 95% CI 1.24–1.32), however this was not risk-adjusted to assess if it remained an independent predictor of graft loss [8]. Molnar and colleagues, looking at the Scientific Registry of Transplant Recipients, found black donor ethnicity was associated with increased risk for all-cause mortality, cardiovascular mortality and graft loss regardless of whether recipients were black or white, although the black-to-black effect was only obtained after numerous risk-adjustments for clinical and transplant-related variables [13].

There are likely to be multi-factorial causes for this increased risk among non-white donors but genetic susceptibility is certainly important. For example, genetic variants (e.g. MYH9 and APOL1) account for a large proportion of the excess risk of end-stage kidney failure observed in African-American compared to non-African American populations [14–17]. We can speculate these kidneys carry their inherent genetic risk for kidney failure with them after kidney implantation into the recipient regardless of their ethnicity. The findings from Reeves-Daniel and colleagues suggest kidneys from African-American deceased donors harboring certain APOL1 risk variants have increased risk for early graft loss failed after kidney transplantation compared to those with zero or one risk variant [18]. However, genetic susceptibility does not fully explain the observed disparity and there is evidence of progressive chornic kidney disease among black patients without high-risk APOL1 variants [19]. However, findings of increased graft loss risk relating to black kidney donors in the United Kingdom cannot be explained by these genetic susceptibilities. APOL1 risk alleles are not found in Europeans but occur in over a third of African Americans [10], although other genetic variants may be at play. In addition, we observed increased risk for graft loss with south Asian donated kidneys and there is little evidence for genetic or biological predisposition to kidney failure in this group. While multi-factorial etiology is likely, our common perception concerning increased risk of progressive chronic kidney disease and end-stage kidney failure among minority ethnics does not actually materialize in systematic reviews of the literature [20].

However, our results should be interpreted in the correct context. For the majority of patients, their mortality risk is significantly better with kidney transplantation compared to remaining on dialysis [21]. One caveat is the observation that minority ethnic patients on dialysis have lower mortality compared to white patients. While this has been frequently reported in the literature and registry data for black patients, more recent analysis from Kucirka and colleagues suggests this phenomenon is only true for older black adults and that mortality is actually increased for black dialysis patients younger than fifty [22]. Subsequent work by Johns and colleagues found this mortality difference for young blacks on hemodialysis was more pronounced in areas of low socioeconomic status and was significantly attenuated in areas of high socioeconomic status [23]. It is possible that lack of adequate insurance coverage is confounding these results (as older adults will be Medicare eligible) and, in the United Kingdom with its universal health coverage, reduced mortality is observed for both black and south Asian dialysis patients in national registry data [24]. While this has never been stratified by age at a population-level, a single-center study of 1,340 patients from Cole and colleagues observed a lower mortality rate for black versus white dialysis patients independent of age, co-morbidity and level of socioeconomic deprivation (although transplantation was not accounted for as a competing risk) [25]. Further studies are warranted to analyze whether the disparate outcomes for minority ethnic dialysis patients is stratified by age as per the United States.

There are several limitations to appreciate in our analysis. It is a retrospective cohort study and comes with the acknowledged limitations of that approach including missing variables, missing data, coding errors, confounding unmeasured variables etc. For our primary outcome of graft failure, longer follow up would provide a better assessment of risk in view of the improved kidney allograft attrition rates seen in the contemporary era of immunosuppression. Due to data limitations, we could only adjust for baseline characteristics but post-transplant adverse events which could influence both risk for graft loss and death. With so many recipient/donor combinations and other factors at play, it is likely our study lacked statistical power to address our particular question regarding ethnicity matching. For deceased donors, we detected and elevated variance inflation factor for matched ethnicity (7.4), suggesting substantial correlation between these two variables, and this has the potential to inflate the standard error and result inn a non-significant hazard ratio (no evidence of correlation in the setting of living kidney donors). Finally, our study was not designed to understand the underlying mechanism of any survival difference relating to donor ethnicity and further work is necessary to shed light on such pathophysiology.

In conclusion, our results confirm the inferior outcomes associated with non-white kidney donors for kidney transplant recipients of any ethnicity in a risk-adjusted model for the United Kingdom population. Contrary to non-renal transplant literature, we did not identify any survival benefits associated with donor-recipient ethnicity matching. Further investigation into mechanisms is warranted to determine if the detrimental effects of non-white donors can be pre-emptively identified and/or attenuated. However, kidney transplantation from any donor ethnicity is likely to still have superior survival outcomes compared to dialysis for patients with end stage kidney failure and should not be discouraged.
